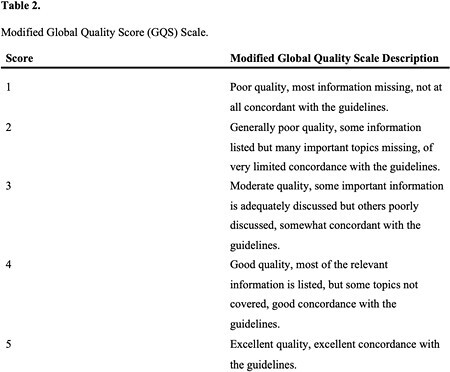# 106 Concordance of ChatGPT with American Burn Association Guidelines on Acute Burns

**DOI:** 10.1093/jbcr/irae036.105

**Published:** 2024-04-17

**Authors:** Jose Antonio Arellano, Sumaarg Pandya, Mario Alessandri-Bonetti, Hilary Liu, Tiffany Jeong, Jenny A Ziembicki, Francesco Egro

**Affiliations:** University of Pittsburgh Medical Center, Pittsburgh, PA; University of Pittsburgh Medical Center, Pittsburgh, PA; University of Pittsburgh Medical Center, Pittsburgh, PA; University of Pittsburgh Medical Center, Pittsburgh, PA; University of Pittsburgh Medical Center, Pittsburgh, PA; University of Pittsburgh Medical Center, Pittsburgh, PA; University of Pittsburgh Medical Center, Pittsburgh, PA

## Abstract

**Introduction:**

Burn injuries often require immediate assistance and specialized care for optimal management and outcomes. The emergence of accessible artificial intelligence technology has just recently started being applied to healthcare decision-making and patient education. However, its role in clinical recommendations is still under scrutiny. This study aims to evaluate whether ChatGPT general expertise on domestic burn management and appropriate responses to commonly asked questions regarding acute burn injuries compared to the American Burn Association guidelines.

**Methods:**

Twelve commonly asked questions were formulated by a fellowship-trained burn surgeon to address the American Burn Association’s recommendations on burn injuries, management, and patient referral. These questions were prompted into ChatGPT, and each response was compared with the aforementioned guidelines, the gold standard for accurate and evidence-based burn care recommendations. Three burn surgeons independently evaluated the appropriateness and comprehensiveness of each ChatGPT response based on the guidelines according to the modified Global Quality Score scale.

**Results:**

The average score for ChatGPT-generated responses was 4.56 ± 0.65, indicating the responses were exceptional quality with the most important topics covered and in high concordance with the guidelines. Overall, 58% of the questions were focused on first aid and management of acute burns, while 42% focused on patient referral criteria. Of the twelve questions asked, two (16.7%) ChatGPT-generated responses scored a perfect 5.00 ± 0.00, demonstrating perfect concordance with the ABA guidelines. Nine (75.0%) ChatGPT answers between good and excellent quality, with average GQS scores ranging from 4.33 to 4.67. One (8.3%) response describing chemical burn referral recommendations scored a 3.33 ± 0.58, indicating somewhat concordance with the ABA guidelines.

**Conclusions:**

This initial comparison of ChatGPT-generated responses and the American Burn Association guidelines demonstrates that ChatGPT can accurately and comprehensibly describe appropriate treatment and management plans for acute burn injuries. We foresee that ChatGPT may play a role as a complementary tool in medical decision-making and patient education, having a profound impact on clinical practice, research, and education.

**Applicability of Research to Practice:**

This research enhances burn injury care and education by using ChatGPT as a complementary tool to provide accurate guidance to help aid healthcare decisions, patient education, and medical training.